# A Novel Frame-Selection Metric for Video Inpainting to Enhance Urban Feature Extraction

**DOI:** 10.3390/s24103035

**Published:** 2024-05-10

**Authors:** Yuhu Feng, Jiahuan Zhang, Guang Li, Ren Togo, Keisuke Maeda, Takahiro Ogawa, Miki Haseyama

**Affiliations:** 1 Graduate School of Information Science and Technology, Hokkaido University, Sapporo 060-0814, Japan; feng@lmd.ist.hokudai.ac.jp (Y.F.); zhang@lmd.ist.hokudai.ac.jp (J.Z.); 2 Education and Research Center for Mathematical and Data Science, Hokkaido University, Sapporo 060-0812, Japan; guang@lmd.ist.hokudai.ac.jp; 3 Faculty of Information Science and Technology, Hokkaido University, Sapporo 060-0814, Japan; togo@lmd.ist.hokudai.ac.jp (R.T.); ogawa@lmd.ist.hokudai.ac.jp (T.O.); 4 Data-Driven Interdisciplinary Research Emergence Department, Hokkaido University, Sapporo 060-0813, Japan; maeda@lmd.ist.hokudai.ac.jp

**Keywords:** video frame selection, video inpainting, quality evaluation

## Abstract

In our digitally driven society, advances in software and hardware to capture video data allow extensive gathering and analysis of large datasets. This has stimulated interest in extracting information from video data, such as buildings and urban streets, to enhance understanding of the environment. Urban buildings and streets, as essential parts of cities, carry valuable information relevant to daily life. Extracting features from these elements and integrating them with technologies such as VR and AR can contribute to more intelligent and personalized urban public services. Despite its potential benefits, collecting videos of urban environments introduces challenges because of the presence of dynamic objects. The varying shape of the target building in each frame necessitates careful selection to ensure the extraction of quality features. To address this problem, we propose a novel evaluation metric that considers the video-inpainting-restoration quality and the relevance of the target object, considering minimizing areas with cars, maximizing areas with the target building, and minimizing overlapping areas. This metric extends existing video-inpainting-evaluation metrics by considering the relevance of the target object and interconnectivity between objects. We conducted experiment to validate the proposed metrics using real-world datasets from Japanese cities Sapporo and Yokohama. The experiment results demonstrate feasibility of selecting video frames conducive to building feature extraction.

## 1. Introduction

In today’s increasingly digitalized society, software and hardware development for capturing video data has made it possible to gather and analyze large amount of video data extensively [1]. This trend has sparked interest in extracting the environment and object information from video data, such as buildings and urban streets [2]. The structure and features of buildings are fundamental components of urban cities and contain rich information relevant to people’s daily lives. Extracting features from various urban buildings and streets and modeling and integrating them with technologies such as VR [3,4,5] and AR [6,7] can lead to more intelligent and personalized urban public services [8,9]. For example, city traffic flow can be better managed to realize intelligent traffic management by analyzing the location of buildings and traffic patterns to model a three-dimensional (3D) map [10,11,12]. This enables smart traffic light control, optimized traffic routes and improved city traffic flow. Therefore, extracting object features from video data has significant implications for urban planning, safety monitoring and environmental management [13].

However, dynamic objects such as pedestrians or vehicles inevitably appear in the video frame when collecting videos of urban streets and buildings for the purposes above, causing interference with the feature extraction of the target objects [14,15]. To address this issue, we incorporated video-inpainting technology. Video inpainting is an advanced video-restoration method that involves meticulously reconstructing missing or damaged segments within a video sequence to meet real-world demands for enhanced visual content [16,17]. It aims to fill the “corrupted” regions with plausible and coherent content throughout video clips [18,19,20]. With the development of computer vision [19,20,21], several video-inpainting methods are constantly being proposed. To improve the quality of inpainting, spatial structure and temporal consistency in the video data need to be considered. However, the original video data exhibit uneven quality levels, introducing complexities in certain video scenarios and decreasing the quality of inpainting results. In such cases, inpainting on each frame may be performed independently. However, this approach often yields time-inconsistent repair results and may give rise to severe artifacts, as noted in the literature [22]. To tackle the above problem, an effective approach is to introduce the optical flow-based video-inpainting method [16,23] that utilizes optical flow information to eliminate occlusions that may affect feature extraction in the video. Optical flow refers to the motion pattern of pixels in a scene over time, which can be used to describe the motion relationship between neighboring frames in an image sequence [24]. Flow-based video-inpainting methods try to infer the content of missing frames and restore the entire video sequence by analyzing the motion information between adjacent frames, thus naturally maintaining temporal consistency [25]. Therefore, we aim to employ this video-inpainting method to eliminate objects that may cause interference from the video data, leaving only the target object for feature extraction.

Moreover, the shape of the target building in each video frame varies [26]. To ensure the quality of the extracted feature, careful selection of frames for processing is essential. Intuitively, we aim to identify frames characterized by a minimal area of occlusions and a maximal area occupied by the buildings of the feature extraction target. Finding an evaluation metric for selecting video frames becomes crucial in this scenario. Therefore, it is essential to obtain a metric that considers three key points: minimizing the area of occlusions before the inpainting process, maximizing the area with the target building and minimizing the overlapping area between the above two objects. Remarkably, previous studies have not proposed tasks or evaluation metrics for this specific purpose. Since our aim is to introduce the video-inpainting method to process the video and then select the appropriate video frames, we consider two factors: whether noise is generated after inpainting processing, resulting in loss of image content, and whether the target building in the image has clear outlines and is easy to extract detailed features. Based on the above two factors, we can evaluate whether the frame is suitable for feature extraction of the target object. For the first point, there are currently many quality-evaluation metrics for images or video frames, including NIQE [27], BRISQUE [28] and PIQE [29]. Since the goal of the video-inpainting method is to modify the image content as required, there is no original reference object that can be used as a baseline ground truth. Therefore, we aim to use the no-reference evaluation metrics [30,31], such as NIQE, to assess image quality. These metrics calculate statistical features, including mean, standard deviation and gradient, providing a quantitative evaluation of the quality of the modified images. However, these metrics only focus on objectively comparing the spatial structure information and characteristics of the processed data without considering the relevance of objects in image content.

**Figure 1:**
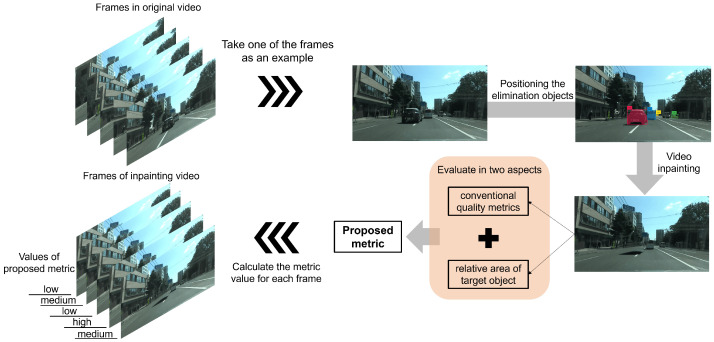
Concept of the proposed metric of selecting video frames for extracting the features of the target object. The proposed metric takes into account the quality of frames after inpainted occlusions (set as “car” in this paper) and the relative area of the target object for feature extraction. Based on the conventional image quality-evaluation metric, the correlation between target object areas is calculated so that the relevance between targets in the image is also taken into consideration.

In this paper, we propose a novel evaluation metric that takes into account the quality of video inpainting and the relevance of the target object, such as buildings, to identify the most suitable frame for extracting the target features. Figure 1 shows the underlying concept of the proposed frame-selection metric. Before using this evaluation metric to select appropriate video frames, we initially adopted the video-inpainting method to eliminate occlusions from video data. Specifically, we introduce the end-to-end framework for the flow-guided video-inpainting (E^2^FGVI) [18] method. By designing three modules in this method that operate in close collaboration, the over-reliance on intermediate results of previously independent systems is alleviated and can work more efficiently. Furthermore, we used the proposed evaluation metric to select frames suitable for extracting target object features. This video frame-selection metric extends existing video-inpainting-evaluation metrics. The extension involves calculating the relevance of the target object areas in the images, considering the interconnectivity between objects. We conduct experiments using several real-world datasets captured in specific scenarios to validate the proposed method. These datasets were collected from Japanese cities in Sapporo and Yokohama. The results provide a feasible method for selecting video frames that are conducive to building feature extraction.

The main contributions of this study are summarized as follows.

- To better extract target objects and features from video data, we propose a novel evaluation metric for screening suitable video frames based on video inpainting.
- We explicitly introduce the calculation of the correlation between the target and surrounding objects, expanding the previous video-inpainting-evaluation metrics to screen suitable video frame data better.

The remainder of this paper is organized as follows. Section 2 is a brief overview of the related works. Section 3 presents a detailed description of the proposed novel evaluation metric. The experimental results are presented in Section 4, where we provide qualitative and quantitative results of the proposed method. Section 5 discusses the implications of our findings and the limitations associated with our study. Finally, Section 6 presents the conclusion.

## 2. Related Work

### 2.1. Video Inpainting

Video-inpainting methods can be broadly divided into three approaches: 3D convolution-based, attention-based and flow-based methods. Each approach leverages distinct strategies to address the challenges of reconstructing missing information in video sequences.

**Three-dimensional convolution-based method.** Three-dimensional convolution exploits the spatiotemporal cube structure of video data. It captures the continuity and temporal relationships within the video by simultaneously considering the temporal and spatial dimensions [32,33]. Chang et al. [34] proposed a learnable gated temporal shift module to process spatial and temporal relationships in video sequences. By introducing a gating mechanism, the module can learn and adjust time-shift operations adaptively to capture dynamic features in videos better. However, this new module may increase the computational complexity of the network, resulting in poor performance when running in some resource-constrained environments. A proposal-based video-inpainting approach was introduced in [35]. This method employs a 3D convolutional network to generate an initial inpainting result and subsequently refines it by matching and fusing a set of candidate regions. This approach effectively leverages the spatial and nonlocal information over time. However, it is worth noting that this method may encounter challenges in accurately capturing complexities present in scenes characterized by rapid changes or intricate dynamics. The inherent limitations may hinder its effectiveness in such dynamic and intricate visual content.

**Attention Mechanisms in Video Inpainting.** Attention mechanisms weigh the importance of different regions in video frames, allowing more focused processing of the inpainting task [20,36]. In this way, they can flexibly focus on inpainting regions, reducing computational complexity and more effectively handling non-continuous and rapidly changing objects in complex scenes [37]. Lee et al. [38] trained a deep neural network (DNN)-based framework that copies the corresponding content in the reference frame and pastes it into the target frame. They included an alignment network that computes affine matrices between frames for alignment, allowing the network to obtain information from more distant frames for robustness. However, this method of focusing on local features sometimes leads to overlooking important timing information for tasks that require global context. Zeng et al. [17] proposed a deep generation model that uses multi-head and multi-layer space-time transformers to perform attention matching on spatial blocks of different scales, thereby finding relevant content in the space and time dimensions to fill in the missing areas and can generate both video-restoration results with perceptual quality and spatial-temporal consistency. However, this method may cause distortion or blur when restoring detailed structures, such as elongated or small objects. This may affect the quality of the repair results. Despite significant progress in recent years, the design of attention mechanisms must be carefully balanced to avoid overly focusing on certain areas while ignoring other important information.

**Optical Flow Estimation and Propagation.** Some methods use optical flow estimation techniques to predict the motion of missing areas for inpainting, which can effectively handle motion in videos [18,39]. Optical flow refers to the movement pattern of pixels in a scene that changes over time [23]. It can be used to describe the movement relationship between adjacent frames in an image sequence. Wang et al. [40] introduced an enhanced deformable convolutional network video-inpainting method that incorporates a feature alignment module that includes the crucial step of optical flow estimation. This module serves the purpose of spatially and temporally aligning input frame features to enhance the capture of motion and deformation. In a similar approach, Chan et al. [41] employed bidirectional propagation and optical flow alignment in their video super-resolution (VSR) method, BasicVSR. Optical flow is instrumental in spatially transforming and aligning features from different frames, which are subsequently concatenated and fused [25,42]. The resulting output image is generated through an upsampling module. It is worth noting that optical flow methods are amenable to training in an unsupervised or weakly supervised manner [43,44,45]. Consequently, video-inpainting methods based on optical flow prove advantageous in capturing object motion information within a video through optical flow estimation.

### 2.2. Evaluation Metrics of Inpainting Video

Since the video-inpainting method aims to modify the image content as required, there is no original image that can be used as a baseline ground truth. Therefore, we tend to use the no-reference evaluation metrics. No-reference image quality-evaluation metrics are used to assess the quality of an image without comparing it to a reference or original image. These metrics are particularly useful when a reference image is not available or when evaluating images in real-world scenarios where the original image might be unknown or inaccessible [46,47]. No-reference image quality-evaluation metrics typically make use of various properties of images to perform calculations. Some of the key properties and features commonly used in these metrics include spatial information, color information, structural information and statistical measures [48,49,50].

**NIQE** [27] is a natural image-based quality-assessment method for measuring the perceived quality of images without reference to ground truth. NIQE primarily focuses on naturalness and the extent of distortion in images, with lower scores indicating better image quality. In the context of video inpainting, NIQE can be used to evaluate the perceptual quality of inpainted video frames, providing researchers with a convenient and reliable tool. The calculation is defined as follows:

(1) $$\mathrm{NIQE}=c_{1}\cdot \mu +c_{2}\cdot \sigma +c_{3}\cdot \left(\frac{\mu }{\sigma }\right)+c_{4}\cdot \frac{1}{N}\sum_{i=1}^{N}\left(\frac{\partial^{2}\mu }{\partial x_{i}^{2}}+\frac{\partial^{2}\mu }{\partial y_{i}^{2}}\right),$$

where μ represents the mean of the image, σ represents the standard deviation of the image, $\frac{\mu }{\sigma }$ represents the ratio of mean to standard deviation, *N* represents the total number of pixels in the image, $\frac{\partial^{2}\mu }{\partial x_{i}^{2}}$ and $\frac{\partial^{2}\mu }{\partial y_{i}^{2}}$ represent the second derivatives of the mean in the horizontal and vertical directions, respectively, and $c_{1},c_{2},c_{3},c_{4}$ are constants. This equation describes the natural properties of an image, evaluating its quality based on statistical information, such as mean, standard deviation and gradients.

**BRISQUE** [28] is a no-reference image spatial quality-evaluation metric that concentrates on visual quality defects, such as distortion, artifacts and false colors within images. BRISQUE assesses perceptual quality by computing natural features in images, making it applicable for evaluating visual quality in video-inpainting scenarios. The calculation is defined as follows:

(2) $$\mathrm{BRISQUE}=\sum_{i=1}^{N}\alpha_{i}\cdot f_{i}\left(\cdot \right),$$

where *N* represents the number of blocks into which the image is divided, $\alpha_{i}$ represents the weight of the *i*-th feature and $f_{i}\left(\cdot \right)$ represents the *i*-th feature function corresponding to the *i*-th statistical feature. BRISQUE relies on a large number of local image features and statistical information obtained through the analysis of image blocks.

**PIQE** [29] is a perceptual image quality-evaluation metric that incorporates human visual perception characteristics, including brightness, contrast and color. When assessing inpainted video frames, PIQE offers a comprehensive evaluation of perceptual quality, providing researchers with insights into the overall visual effects of the inpainting results. The calculation is defined as follows:

(3) $$\mathrm{PIQE}=\alpha_{1}\cdot C+\alpha_{2}\cdot M+\alpha_{3}\cdot S,$$

where *C* represents colorfulness, *M* represents sharpness, *S* represents contrast and $\alpha_{1},\alpha_{2},\alpha_{3}$ are weighting coefficients. Colorfulness, sharpness and contrast are important visual features for image quality. PIQE combines these features, adjusting their contributions with weights to evaluate the perceived quality of an image comprehensively.

The application of these no-reference evaluation metrics in video inpainting offers researchers objective means of quantifying inpainting effectiveness, contributing to the advancement and optimization of this field.

## 3. Frame-Selection Metric for Video Inpainting

**Figure 2:**
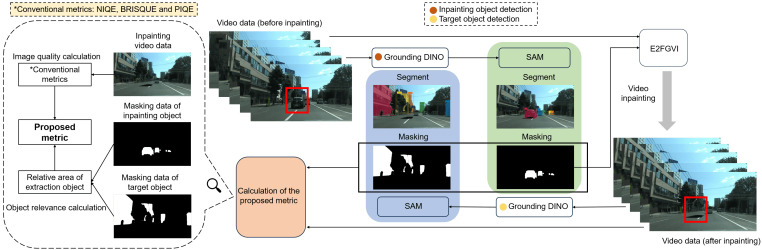
Overview of the calculation of the proposed metric. We extract the position coordinate of the “car” using the Grounding DINO model and input it into the SAM model for more accurate segmentation and masking results. Subsequently, the original video data and the “car” masking data are fed into the E^2^FGVI model for video inpainting. In the obtained video, the outline of the target object (“building”) becomes clearer. Following this, we also employ the Grounding DINO and SAM models to obtain masking data for the “building”. This, along with the previously obtained “car” masking and video data after inpainting, is used to calculate the proposed metric.

To reduce the impact of occlusions on feature extraction, we employ video inpainting on the original video data and then assess frames while considering semantic content. As shown in Figure 2, we employ the advanced E^2^FGVI method and integrate the Grounding DINO object detector with the segment anything model (SAM) to provide mask data for E^2^FGVI. We use the Grounding DINO detector to obtain object coordinates, with which SAM can precisely generate mask data through segmentation. After applying E^2^FGVI to the obtained masks, we evaluate frame quality and object correlation to select the optimal frame for feature extraction.

### 3.1. Generation of Mask Data

Grounding DINO initially identifies the coordinates of the target object for elimination using a dual encoder–single decoder architecture. It comprises image and text backbones for feature extraction, a feature enhancer for fusing image and text features, a language-guided selection module for initializing queries and a cross-modality decoder for refining box coordinates [51]. The feature enhancer module facilitates cross-modality feature fusion, and the language-guided query-selection module selects queries from image features. These queries are input into a cross-modality decoder that updates and probes desired features. The decoder’s output queries predict object boxes and extract corresponding phrases. In this study, we used the Grounding DINO model with “car” as the keyword to derive the boundary box corresponding to the approximate position of objects in each frame image, being used to generate mask data of the corresponding area in the next step.

These boundary boxes indicate the target object’s position in the image but lack detailed target outline information, posing challenges for accurate calculation of the relative area in subsequent screening. To overcome this limitation, we use the obtained boundary box data to locate the target precisely using the SAM model. The spatial attention masking method is then applied to accurately mask the corresponding target in the SAM video frame. Built on a vision transformer with real-time performance optimizations, the masking method considers sparse and dense prompts. The mask decoder efficiently maps the embeddings and an output token to a mask. After two blocks, the image embedding is upsampled and a multilayer perceptron maps the output token to a dynamic linear classifier. Through this process, the mask data for the “car” in the frames were obtained to guide the video-inpainting model in eliminating the “car” area in the frames.

### 3.2. Inpainting of Certain Objects

To address the removal of specific objects within the masked area of video frames, we introduced the E^2^FGVI method. For a video sequence $X^{t}$ that can be defined as $\left\{X^{t}\in R^{H\times W\times 3}|t=1,2,...,T\right\}$ with a sequence length of *T* and corresponding frame-wise binary masks, we aim to synthesize faithful content that maintains consistency in the spatial and temporal dimensions within the corrupted (masked) areas. The methodology begins with a context encoder for encoding all corrupted frames into lower-resolution features to enhance computational efficiency in subsequent processing [18]. Subsequently, we employ a flow completion module to extract and complete the optical flow between local neighbors. The completed optical flow assists in aligning features extracted from local neighbors, facilitating bidirectional propagation. Furthermore, content hallucination is performed using multi-layer temporal focal transformers, combining propagated local neighboring features with non-local reference features. Finally, a decoder is used to upscale the filled features, reconstructing them into a final video sequence $\left\{{\hat{Y}}^{t}\in R^{H\times W\times 3}|t=1,2,...,T\right\}$. Using the mask data for “car” as input, we employ the E^2^FGVI model to eliminate the content related to “car” in the original video data.

It is worth noting that the evaluation of the image quality of processed frames requires a comprehensive analysis of image data from diverse perspectives. This evaluation includes assessing whether the frame is conducive to extracting target object features. The factors considered include the relative area size of the target object within the image and the clarity of its outline. To facilitate this evaluation, it is essential to obtain area data for the “elimination target” and “feature extraction target”. However, a potential challenge arises in scenarios where the feature extraction target (building) may encounter obstruction from cars in the original video data, resulting in the blurring of the building’s outline. To address this issue, we perform masking after video inpainting as shown in Algorithm 1. This step aims to clearly delineate the outline of the building and calculate the relative area accurately. Following the acquisition of the video frame after inpainting the “car” object, we iterate through the processing steps outlined in Section 3.1. In this iteration, we replace the keyword with “building” to conduct a similar analysis for the desired feature extraction target. Finally, we obtained the mask data of the “building”.
**Algorithm 1** Generate mask data and perform video inpainting1:**procedure**2:    Input: meta video data $v_{\mathrm{meta}}$ with *N* frames3:    **for** each frame $F_{i}(i=1$ to N) in $v_{\mathrm{meta}}$ **do**4:        # Generate the mask data of “car”5:        $m_{\mathrm{car}}^{i}\leftarrow \mathrm{GroundingDINO}\left(F_{i}\right)$6:    **end for**7:    $M_{\mathrm{car}}={\left[m_{\mathrm{car}}^{i}\right]}_{i=1}^{N}$8:    # Eliminate “car” from video data9:    $v_{\mathrm{without}}\_\mathrm{car}\leftarrow E^{2}\mathrm{FGVI}\left(v_{\mathrm{meta}},M_{\mathrm{car}}\right)$10:    **for** each frame $F_{i}(i=1$ to N) in $v_{\mathrm{without}}\_\mathrm{car}$ **do**11:        # Generate the mask data of “building”12:        $m_{\mathrm{building}}^{i}\leftarrow \mathrm{GroundingDINO}\left(F_{i}\right)$13:    **end for**14:    $M_{\mathrm{building}}={\left[m_{\mathrm{building}}^{i}\right]}_{i=1}^{N}$15:    **return** $v_{\mathrm{without}}\_\mathrm{car}$, $M_{\mathrm{car}}$, $M_{\mathrm{building}}$16:**end procedure**

### 3.3. Derivation of Novel Metric and Frame-Selection Scheme

After the above processing, we can gain the mask data for the object “car” in the original video data as A, the video frame after inpainting the “car” using E^2^FGVI defined as B and the mask data for the object “building” in B as C. We first evaluate the frame image quality after inpainting from the aspect of image data by calculating traditional image quality-evaluation indicators. Specifically, we use three metrics: NIQE, BRISQUE and PIQE.

We also need to evaluate whether the frame is suitable for feature extraction of the target object from the semantic level perspective, i.e., the relative area size of the target object in the image and whether the outline is clear. Our starting point is that we hope that the area where people or vehicles appear in the selected frame is as small as possible to highlight the target building as much as possible. The part containing the building needs to be as large as possible, whereas the overlapping area of the above two areas is minimal. Therefore, as shown in Algorithm 2, taking this “elimination target” (car) and “feature extraction target” (building) as an example, we set the total area occupied by the two in the image to ${\mathrm{Area}}_{\mathrm{all}}$ and defined as

(4) $${\mathrm{Area}}_{\mathrm{all}}=\mathrm{Area}\left(A\right)+\mathrm{Area}\left(C\right)-O\left(A,C\right),$$

where Area(·) and O(·) represent the area and the overlapping area of two objects. Consequently, we define the ratio of the area of the “elimination target” as ${\mathrm{Area}}_{r}\left(A\right)$, ratio of the area of the “feature extraction target” as ${\mathrm{Area}}_{r}\left(C\right)$ and the ratio of overlap of the two objects as $O_{r}\left(A,C\right)$. The specific calculation method is as follows:

(5) $$\begin{array}{cc} {\mathrm{Area}}_{r}\left(A\right) & =\mathrm{Area}\left(A\right)/{\mathrm{Area}}_{\mathrm{all}}, \end{array}$$

(6) $$\begin{array}{cc} {\mathrm{Area}}_{r}\left(C\right) & =\mathrm{Area}\left(C\right)/{\mathrm{Area}}_{\mathrm{all}}, \end{array}$$

(7) $$\begin{array}{cc} O_{r}\left(A,C\right) & =O\left(A,C\right)/{\mathrm{Area}}_{\mathrm{all}}. \end{array}$$

Next, we define the parameter $R_{\mathrm{iou}}\left(A,C\right)$ that comprehensively considers the relationship between the two areas as follows:

(8) $$R_{\mathrm{iou}}\left(A,C\right)=\frac{1}{{\mathrm{Area}}_{r}\left(A\right)+O_{r}\left(A,C\right)+1}+{\mathrm{Area}}_{r}\left(C\right),$$

Specifically, the smaller the area ratio of the occlusion (${\mathrm{Area}}_{r}\left(A\right)$), the less interference there is in the feature extraction process. Similarly, a smaller overlapping area ratio ($O_{r}\left(A,C\right)$) between the occlusion and the target object indicates a clearer outline of the target object in the frame. Finally, a larger area ratio of the target object (${\mathrm{Area}}_{r}\left(C\right)$) implies that it is easier to capture detailed features in the frame. Therefore, during the calculation, we add ${\mathrm{Area}}_{r}\left(A\right)$ and $O_{r}\left(A,C\right)$ and take the reciprocal. ${\mathrm{Area}}_{r}\left(C\right)$ is treated as a separate component and added to the fractions. Besides, to avoid the situation where ${\mathrm{Area}}_{r}\left(A\right)+O_{r}\left(A,C\right)$ equals 0, which occurs when the frame does not initially contain occlusion and thus the calculation of the parameter $R_{\mathrm{iou}}$ cannot proceed, we introduce the term +1 to the denominator ensuring the smooth progression of the calculation process. Moreover, the smaller the score of the above three conventional image quality-evaluation metrics, the better the perceived quality. Thus, we define the new frame-selection metric as follows:

(9) $$q=\mathrm{Sigmoid}(\frac{1}{metrics}+R_{\mathrm{iou}}\left(A,C\right)),$$

where metrics∈{NIQE,BRISQUE,PIQE}. Since the conventional metrics NIQE, BRISQUE and PIQE are all smaller values indicating better image quality, to match with $R_{\mathrm{iou}}$, we take the inverse of the conventional metrics for calculation and map the sum of the two to the interval from 0 to 1 by using the sigmoid function for easy comparison. The definition of q includes image quality evaluation and feature extraction target relative area. It evaluates the image quality of the processed frame from the image data perspective. It evaluates whether the frame is suitable for the target from the semantic level perspective. Feature extraction of objects. The higher the value of q, the more suitable the frame is for extracting features of the set target.

The proposed metric considers both image quality and object relevance during the calculation. Frames obtained through the selection with higher values of q can be considered to have a higher comprehensive level in both aspects. In this way, the frames whose image content is most suitable for extracting target object features are obtained from the video, which can effectively support and assist a series of downstream tasks. Taking “buildings” as the target in this study, extracting features from city buildings and streets and subsequently modeling and integrating them with technologies such VR and AR holds significant importance. This approach contributes to more intelligent and humanized urban public services, impacting urban planning, security monitoring and environmental management.
**Algorithm 2** Calculation of the proposed metric1:**procedure**2:    Input: $v_{\mathrm{without}}\_\mathrm{car}$, $M_{\mathrm{car}}$, $M_{\mathrm{building}}$3:    **for** each frame $F_{i}(i=1$ to N) in $v_{\mathrm{without}}\_\mathrm{car}$ **do**4:        # Calculate the ratio of the mask area to the each frame5:        ${\mathrm{Area}}_{r}\left(m_{\mathrm{car}}^{i}\right)\leftarrow \mathrm{the}\;\mathrm{ratio}\;\mathrm{of}\;m_{\mathrm{car}}^{i}\;\mathrm{in}\;F_{i}$6:        ${\mathrm{Area}}_{r}\left(m_{\mathrm{building}}^{i}\right)\leftarrow \mathrm{the}\;\mathrm{ratio}\;\mathrm{of}\;m_{\mathrm{building}}^{i}\;\mathrm{in}\;F_{i}$7:        $O_{r}\left(m_{\mathrm{car}}^{i},m_{\mathrm{building}}^{i}\right)\leftarrow \mathrm{the}\;\mathrm{ratio}\;\mathrm{of}\;\mathrm{the}\;\mathrm{overlapping}\;\mathrm{area}\;\mathrm{between}\;\mathrm{two}\;\mathrm{masks}\;\mathrm{in}\;F_{i}$8:        # Comprehensively considers the relationship between the two masks areas9:        $R_{\mathrm{iou}}\left(m_{\mathrm{car}}^{i},m_{\mathrm{building}}^{i}\right)\leftarrow \frac{1}{{\mathrm{Area}}_{r}\left(m_{\mathrm{car}}\right)+O_{r}\left(m_{\mathrm{car}},m_{\mathrm{building}}\right)+1}+{\mathrm{Area}}_{r}\left(m_{\mathrm{building}}\right)$10:        # No-reference image quality evaluation11:        $metrics\in \left\{\mathrm{NIQE},\mathrm{BRISQUE},\mathrm{PIQE}\right\}\leftarrow \mathrm{calculate}\;\mathrm{the}\;\mathrm{image}\;\mathrm{quality}\;\mathrm{of}\;F_{i}$12:        # The proposed selection metric13:        $q\leftarrow \mathrm{Sigmoid}(\frac{1}{metrics}+R_{\mathrm{iou}}\left(m_{\mathrm{car}}^{i},m_{\mathrm{building}}^{i}\right))$14:    **end for**15:    **return** q16:**end procedure**

## 4. Experiments

In this section, we explain the experiments on the proposed frame-selection metric. We also introduce the relevant settings used in the experiment in Section 4.1 and explain the results of the experiment in Section 4.2.

### 4.1. Condition

**Dataset.** We conducted experiments on real-world datasets obtained from a style of street scene shot along the road by a vehicle-mounted camera ZED2i equipped with dual 4M pixels sensors with 2-micron pixels. The video output resolution is side-by-side 2×(1920×1080) with 30 frames per second. During the filming, the camera-mounted vehicle was traveling at a speed of approximately 20 km/h. Specifically, the datasets are sourced from Japanese cities, including Sapporo, Yokohama and shin Yokohama. For convenience of description, we call the video datasets Sapporo, Yokohama and shin Yokohama, respectively, for they were shot in the corresponding cities or regions. All these video datasets were shot at a street scene using a fixed-position camera located in front of the car. In the experiment, we selected two clips from the above three datasets. Each clip contains 50 frames of images intercepted from a random moment in the meta video data.

In this paper, we first propose a frame-selection metric that considers the quality of the inpainting video and the relative area occupied by the target object. Therefore, the models we used in the experiments, such as Grounding DINO, SAM and E^2^FGVI, followed the parameter settings in the original paper without additional training. For Grounding DINO, we use six feature enhancer layers in the feature enhancer module and the cross-modality decoder comprises six decoder layers [51]. In SAM’s transformer structure, we used eight heads in all attention layers [52].

**Evaluation.** We compared the experimental results from two aspects to verify the effectiveness of the proposed selection metric. Specifically, for the same building in the image, we first compare the results of the conventional no-reference image quality-evaluation metric with that of the proposed metric for a certain frame to verify the effectiveness of considering the correlation of objects in the image. Then, we compare the content difference of frames with different calculation results of the proposed metric, especially the outline of the target object and the area it occupies in the image, which is used to determine whether the proposed metric can be effectively screened to obtain frames that meet the feature extraction requirements.

### 4.2. Experimental Results

In this section, we analyze the composition of the proposed metric from the perspective of the validity of the introduction of the concept of object relevance, sensitivity to the nuances of the image content, universality of the improvement for the traditional image quality-evaluation metrics and necessity of introducing the overlap area in the calculation of the object relevance to confirm the validity of the proposed metric.

**Figure 3:**
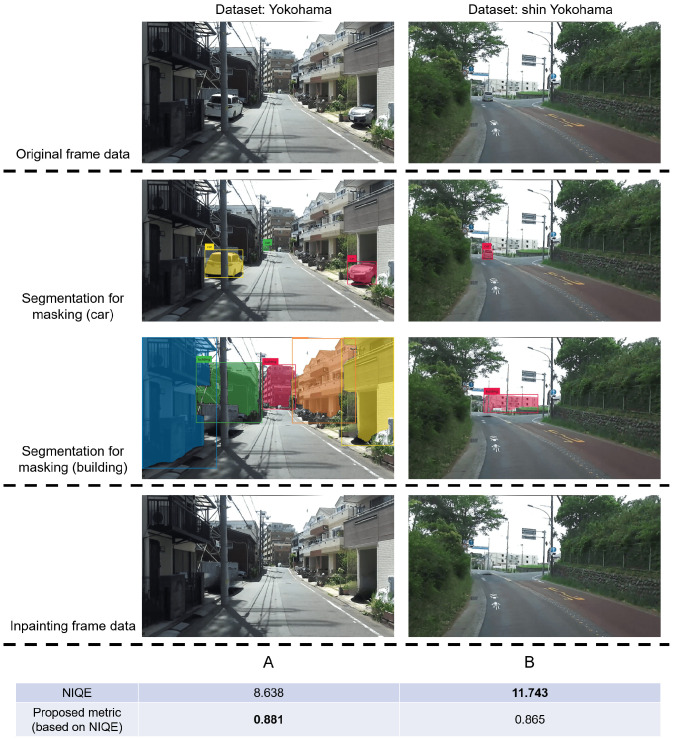
The results in the figure demonstrate the limitations of conventional image quality-evaluation metrics and the effectiveness of introducing object relevance into the proposed metric. The relative area of the building in frame A is larger than that in frame B. Intuitively, frame A is more suitable for extracting building features. However, the results of conventional metrics show that the image quality-evaluation result of frame A is worse than that of frame B. In contrast, the proposed metrics show that frame A is better.

Figure 3 shows a notable disparity in the relative area of buildings between frame A, surpassing that of frame B. The structural content of frame A is more conducive to feature extraction in terms of the suitability for extracting building features from the images. However, the conventional image quality-evaluation metrics indicate that the image quality-assessment for frame A is subpar compared to frame B. The proposed metric achieves higher values for frame A than frame B based on the expected outcome that frame A is more apt for extracting building features. Thus, the effectiveness of the proposed metric is substantiated, especially in incorporating object relevance.

**Figure 4:**
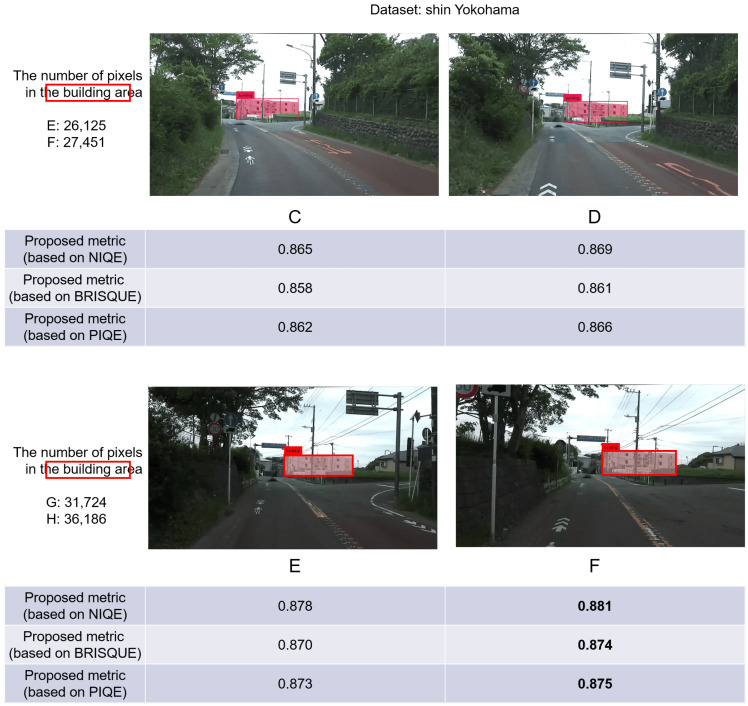
We aim for the area of the buildings in the selected frames to be larger and more conducive to extracting detailed features. This figure illustrates that the area (in pixels) occupied by the buildings in the four frames steadily increases, alongside a rise in the value of the proposed metric. This observation underscores that even minor changes in the area occupied by the target object within the image content of different frames can be quantitatively expressed using the proposed metric.

Figure 4 shows that frames C, D, E and F exhibit minimal temporal separation in the video, leading to highly similar visual content. These four frames in Figure 4 are sequentially increasing in the order of the video timeline. This can be regarded as the camera-mounted vehicle gradually moving forward and approaching the building. In this process, the relative area occupied by the buildings has increased slightly in each frame of D, E and F compared with the previous frame by calculating the number of pixels. The results reveal a sequential increase in the values of the proposed metric for frames C, D, E and F as the area occupied by the buildings in the image expands. These findings underscore that even subtle variations in the relative area occupied by the target object within the image content of different frames can be quantitatively expressed using the proposed metric. Consequently, the discernment of frames more conducive to target object feature extraction becomes feasible, thereby validating effectiveness of the proposed metric. Moreover, Figure 4 facilitates a comparative analysis of the results from the proposed metric computed using three distinct no-reference image quality-evaluation indices. Frames C, D, E and F follow a sequential order along the video timeline. Notably, with a slightly greater relative area of the building, the values of the proposed metric, calculated based on the three conventional image quality-evaluation metrics, exhibit a corresponding increase. This consistency confirms the applicability of the proposed metric’s calculation approach across a range of traditional image quality-evaluation metrics.

**Figure 5:**
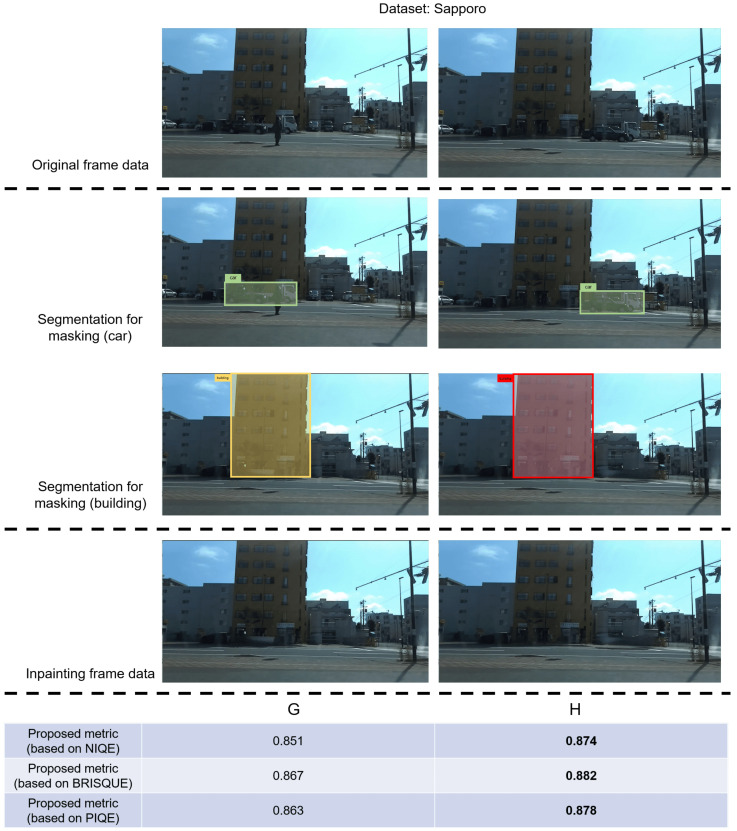
Although the contents of the two frames after inpainting processing are basically the same, the blur left in the background when the occlusion (“car”) is eliminated will still affect the feature extraction of the target object (“building”). The proposed metric integrates the calculation of the overlapping area between the “car” and “building” to discern and exclude frames in which the clarity of the building’s outline is compromised by blur. The results in the figure show that frames with smaller overlapping areas have higher scores.

**Figure 6:**
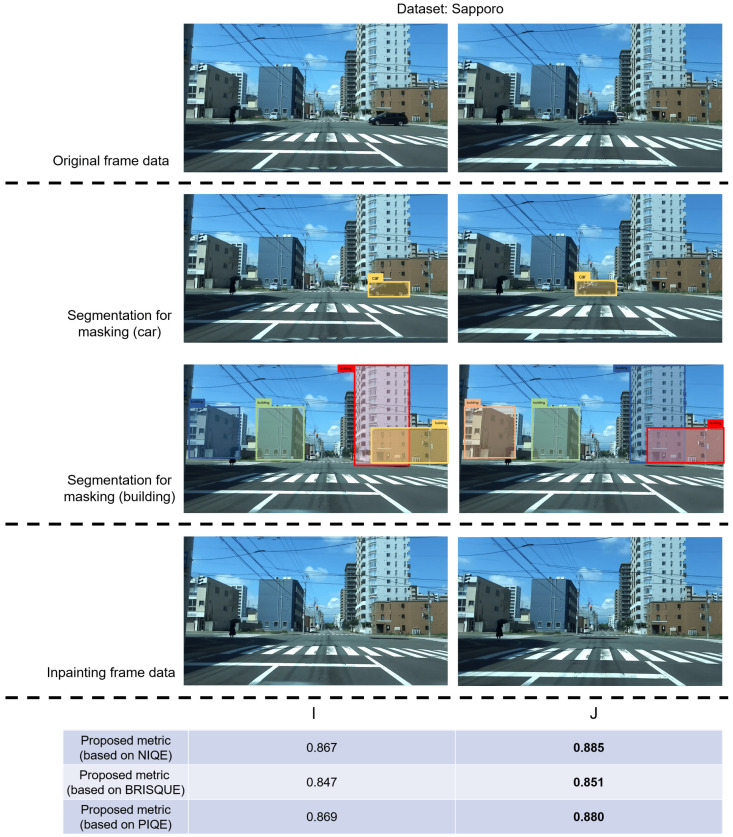
Although the contents of the two frames after inpainting are basically the same. By comparing the original frames, it can be found that after eliminating the “car”-related content, blurring appeared in the processed area, which may affect the recognition of the building’s outline. As shown in this figure, frame K with blurred areas close to buildings has lower scores, whereas frame L with blurred areas far away from buildings has higher scores. The proposed metric can be effective in different scenarios, which further verifies its effectiveness.

**Figure 7:**
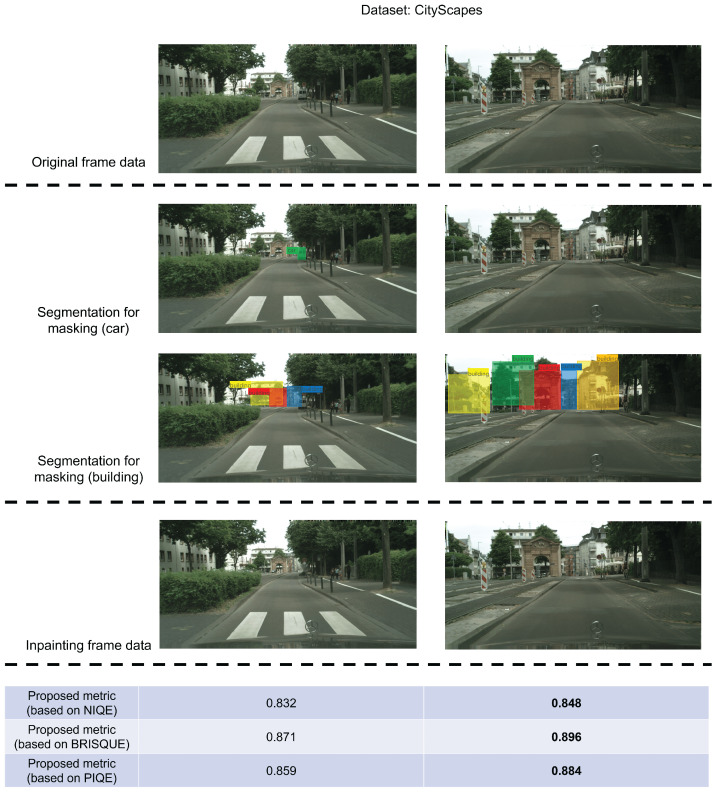
In addition to conducting experiments on private datasets, we also verified the proposed metrics on the public dataset CityScapes. The results in the figure prove the effectiveness of the metrics.

Figure 5 shows that the visual content in frames G and H post-inpainting exhibits substantial similarity; a similar phenomenon is observed in frames I and J in Figure 6. In frames G and I, before undergoing inpainting, cars traverse the front of the building, obstructing the building’s outline in the image. In contrast, in the untreated frames H and J, the cars have almost departed from the building. These figures illustrate that, although the “car” content in the image is mainly eliminated and substituted with background elements through inpainting, the constrained performance of the current video-inpainting method leads to a discernible degree of background blurring in the processed areas. Utilizing frames with such characteristics for extracting building features can impact the overall extraction performance. To address this issue, the proposed metric incorporates the computation of the overlapping area between “car” and “building” to select frames where the building’s outline is influenced by blur during the selection of frames suitable for feature extraction. Figure 5 and Figure 6 show that the score of frames H and J surpasses that of frames G and I, underscoring the effectiveness of the proposed metric. Furthermore, to assess the universality of the proposed metric, in addition to the aforementioned private dataset, we conducted identical experiments using a public dataset, CityScapes. The results presented in Figure 7 demonstrate that the proposed metrics yield higher values in frames where the buildings are more clearly visible, thus validating the effectiveness of the proposed metrics.

In essence, the proposed metric serves as an extension to enhance the performance of conventional image quality-evaluation metrics for specific tasks. Figure 4, Figure 5 and Figure 6 show the results of the proposed metric based on three conventional metrics: NIQE, BRISQUE and PIQE. As shown in these figures, the proposed metric, based on different conventional metrics, accurately identifies frames that are more suitable for feature extraction. This observation validates the effectiveness of the calculation model of the proposed metric across a range of traditional image quality-evaluation indicators.

## 5. Discussion

We have proposed a viable solution within the research domain that focuses on selecting frames from videos for a certain purpose. The innovative evaluation metric introduced for selecting video frames based on video inpainting enhances the ability to select frames suitable for extracting specific target object features. In this section, we discuss the limitations of the existing model and potential avenues for future research.

**Limitations.** The proposed metric is a preliminary conclusion drawn from our comprehensive consideration of the two aspects of this task: the necessity of evaluating the quality of image inpainting and the relevance between the target objects for feature extraction and their surroundings. Therefore, extensive domains remain awaiting exploration and validation to enhance the accuracy of this novel metric. For instance, there is still potential to enhance the accuracy of acquiring mask data prior to the video-inpainting process.

Moreover, the segmentation and mask results depicted in the figure reveal that the current methods sometimes struggle to identify the specified input class accurately. This issue may result in the incapacity to eliminate interfering objects during subsequent video inpainting or may affect the calculation of the relative position of the target object, leading to lower area values. As previously stated, the proposed selection metric considers two factors to comprehensively evaluate the frame’s suitability for extracting target features. However, we directly employed conventional image quality-evaluation metrics at the specific calculation level to assess the image quality after video inpainting. It then evaluates the object relevance in the image by calculating the relative area of the target object and ultimately combines these two aspects.

Finally, the main purpose of this study is to validate the effectiveness of the newly proposed selection metrics. Video inpainting serves as a preprocessing step before calculating the proposed metric and is not restricted to the E^2^FGVI method utilized in this paper. Other inpainting methods with exceptional performance can also serve the same purpose. Therefore, we did not compare the effects of additional methods during the video-inpainting stage in this paper. Consequently, to a certain extent, the impact of the processing results of various video-inpainting methods on the calculation of the proposed metrics was not thoroughly examined. A more in-depth discussion is warranted, exploring aspects such as whether there exists a prioritization in the impact of the two factors on feature extraction. Moreover, during the calculation of the proposed frame-selection metric to obtain a higher value, it remains challenging to discern the predominant influence between the two factors mentioned earlier.

## 6. Conclusions

This paper proposes a novel evaluation metric that considers video-inpainting-restoration quality and the relevance of the target object, such as buildings, to identify the optimal frame for extracting the target features. First, the video data undergoes processing using the video-inpainting method called E^2^FGVI. This method employs three closely modules that operate in close collaboration to enhance efficiency by mitigating over-reliance on intermediate results of previously independent systems. The proposed evaluation metric is then applied to select frames suitable for extracting target object features. This metric extends existing video-inpainting-evaluation metrics by calculating the relevance of target object areas in the images, considering interconnectivity between objects. We conducted experiments on real-world datasets from Japanese cities to validate the effectiveness of the proposed approach. The results demonstrate a practical method for selecting video frames conducive to building feature extraction.

**Future Work.** In future studies, we intend to explore optimizations in the process of obtaining mask data. This involves employing more accurate detection and segmentation methods to generate masks, continuously enhancing the effectiveness of object elimination in subsequent video inpainting. Additionally, considering that this experiment serves as an exploration and preliminary verification of a new research task, the calculation of the relative area of the target object and the clarity of its outline involves only one specific pair, with “buildings” as the target and “cars” as the obstructing objects. Upon validating the effectiveness of the proposed metric through experiments, our future plans involve expanding the scope of relevance calculation to include more objects. This extension may encompass entities such as “pedestrians”, “green belts” and “street lights”.

During the analysis of the results, we observed that certain issues persist when the video-inpainting model E^2^FGVI employed in this experiment eliminates the specified content. These issues include the retention of original content or the introduction of blurred noise in the background. This phenomenon adversely affects the calculation of image quality-evaluation indicators and the segmentation of target objects. Thus, we will continue to optimize video-inpainting methods with enhanced performance. Simultaneously, we will pay close attention to the latest developments in the field of video inpainting and introduce implemented SOTA models for comparison. This ongoing effort is aimed at improving the accuracy and effectiveness of the proposed metric.

Moreover, we plan to conduct extended experiments to validate the significance of the two factors influencing the change in the proposed metric’s value. Additionally, we aim to refine the calculation method of the proposed metric using techniques such as weighting to ensure more reasonable and meaningful results. Finally, in further expansion in the future, the practical application of the proposed method is not limited to select frames mentioned in this article for extracting features. An interesting application is that we can eliminate the occlusion of athletes in image data about sports referees to provide viewers with a better viewing experience.

## Data Availability

Some non-public datasets in this study were provided by Japan Radio Co., Ltd.
